# To Delinquents

**Published:** 1876-11

**Authors:** 


### Editorial and Miscellaneous.

---

*To Delinquents.*—It is always painful for us to call attention, so prominently in our pages, to this class of our readers. We feel assured they do not intend to wrong us, and yet their delays in forwarding the amounts of their subscriptions does wrong us, and injure us, seriously. These delays cause us great, very great, inconvenience, and often we are forced to advance money upon promises made us that these delinquent friends shall not be deprived of the RECORD. We have kept faith with our subscribers. We have always complied with our promises. For nearly six years not one number has failed to come out—not one has been lost, to them. We have adhered strictly to our contract. If the journal has not at all times been prompt in its publication, it has not been the fault of its editors, and, as said above, subscribers have not failed to get every number.

Now, friends, one and all,—you who owe us,—we appeal to you. Save us time, trouble and expense of dunning you through the mails. To write these things is not pleasant, but you know a journal cannot be run without money. Your subscription is but a drop—but drops make up an ocean of debt. Send your money along. Do it at once, and may the gratification and pleasure of performing a duty and doing a good act, be your reward. Let no one refuse. Let the word be—solid column to the front, advance with the cash!

